# EGF and HB-EGF enhance the proliferation of programmable cells of monocytic origin (PCMO) through activation of MEK/ERK signaling and improve differentiation of PCMO-derived hepatocyte-like cells

**DOI:** 10.1186/1478-811X-10-23

**Published:** 2012-08-08

**Authors:** Ayman Hyder, Sabrina Ehnert, Hebke Hinz, Andreas K Nüssler, Fred Fändrich, Hendrik Ungefroren

**Affiliations:** 1 Clinic for Applied Cellular Medicine, UKSH, Campus Kiel, Arnold-Heller Strasse 3, Hs. 18, 24105, Kiel, Germany; 2 BG Unfallklinik Tübingen, Eberhard-Karls Universität Tübingen, Schnarrenbergstr. 95, 72076, Tübingen, Germany; 3 First Department of Medicine, UKSH, Campus Lübeck, Ratzeburger Allee 160, 23538, Lübeck, Germany

**Keywords:** EGF, Hepatocyte-like cells, MEK, Extracellular signal-regulated kinase, Proliferation, Programmable cells of monocytic origin

## Abstract

**Background:**

Hepatocyte-like cells (NeoHepatocytes) generated from a peripheral blood monocyte-derived stem cell-like cell (the PCMO) are a promising alternative for primary hepatocytes in cell transplantation studies to cure liver diseases. However, to be therapeutically effective NeoHepatocytes are needed in large quantities. It was the aim of the present study to investigate i) whether the proportion of actively proliferating NeoHepatocytes can be enhanced by supplementing the PCMO differentiation medium (containing M-CSF, IL-3, and human serum) with either EGF or HB-EGF and ii) which signaling pathway underlies the promitotic effect.

**Results:**

EGF and HB-EGF enhanced cell proliferation of PCMOs as demonstrated by increased expression of cycle control genes (ABL, ANAPC2, CDC2, CDK4, CDK6), phosphorylation of the retinoblastoma protein, and increased PCMO cell numbers after stimulation with EGF or HB-EGF. EGF also raised the number of monocytes expressing the proliferation marker Ki67. PCMOs expressed the EGF receptors EGFR (ERBB1) and ERBB3, and expression of both increased during PCMO generation. Phosphoimmunoblotting of PCMOs indicated that both EGF and HB-EGF activated MEK-1/2 and ERK1/2 in a concentration-dependent fashion with the effect of EGF being more prominent. EGF treatment further decreased expression of p47^phox^ and increased that of Nanog indicating enhanced *de*differentiation and pluripotency, respectively. Treatment with both EGF and HB-EGF resulted in NeoHepatocytes with improved functional parameters.

**Conclusions:**

The results suggested that the addition of EGF or HB-EGF to PCMO differentiation medium superactivates MEK/ERK signaling which then increases both PCMO proliferation, number, and functional differentiation of PCMO-derived NeoHepatocytes.

## Background

Although hepatocyte transplantation is a therapeutic option for end-stage liver diseases, cell material is scarce due to a critical shortage of liver tissues and the lack of protocols that allow maintaining the differentiated hepatocyte phenotype in culture for more than a week. Thus, generation of hepatocyte-like cells from stem cells or stem cell-like cells may represent a promising alternative [1]. One such cell type with inherent stem cell-like features is the human peripheral blood monocyte [2-5]. By initially inducing a process of *de*differentiation we have generated from these cells a more plastic derivative termed “programmable cell of monocytic origin” (PCMO). PCMOs are prone to acquire functional activities of hepatocyte-like cells (NeoHepatocytes) upon stimulation with appropriate differentiation media *in vitro*[2,3], and *in vivo* following transplantation into mice [2].

From the clinical point of view, a major obstacle in cell transplantation is the large amount of cells required to achieve a therapeutic effect in patients. Despite an already large number of cells that can be retrieved from blood products the overall numbers of NeoHepatocytes obtained after the two-step dedifferentiation-differentiation protocol are still low and insufficient. One possibility to increase NeoHepatocyte cell numbers is by inducing the cells to proliferate. This is more likely to be possible at or before the PCMO stage as the NeoHepatocyte differentiation from PCMO is mutually exclusive with proliferation. Indeed, during conversion of peripheral blood monocytes into PCMOs, a process involving *de*differentiation, a fraction of monocytes resume proliferation *in vitro* in response to macrophage-colony stimulating factor (M-CSF), interleukin-3 (IL-3), and human serum [2]. The extent of proliferation however, was not sufficient to substantially increase the overall cellular yield of NeoHepatocytes. If the rate of proliferation and/or the percentage of mitotically active monocytes could be enhanced prior to induction of differentiation, then an increased number of NeoHepatocytes may be obtained, thereby increasing the chance for successful NeoHepatocyte transplantations. Ideally, a modification of the PCMO generation procedure, e.g. by addition of growth-stimulatory factor(s), should not only enhance mitotic activity but also the plasticity of PCMOs in such a way that the resulting NeoHepatocytes become more hepatocyte-like [6]. Interestingly, a subpopulation of human monocytes that proliferates *in vitro* in response to M-CSF has been suspected to be less mature and hence more stem cell-like than other monocytes [7]. Therefore, the identification of growth factor signaling pathways that regulate proliferation of human monocytes may enhance both the quantity and quality of PCMO-derived NeoHepatocytes.

Epidermal growth factor (EGF) is known to induce proliferation in many kinds of cells [8-11] and its receptor is over-expressed in proliferative cells [12]. Another member from the EGF family, the 20–22 kDa glycoprotein Heparin-binding epidermal growth factor (HB-EGF) [13] was also reported to be a potent mitogen for many cell types [14-16]. Human peripheral blood monocytes were shown recently to express a functional EGF receptor (EGFR) [17,18], while the EGF receptors c-ERBB2, 3 and 4 have not been studied. However, a link between EGF or HB-EGF and proliferation in monocytes has never been investigated. Analysis of the mechanism of receptor tyrosine kinase activation in monocytes may identify soluble factors that control PCMO self renewal. The present study aimed to investigate the expression and the activity of the epidermal growth factor receptor (ERBB) family in human peripheral monocytes and the role of EGF and HB-EGF on the outcome of PCMO generation and the subsequent differentiation into NeoHepatocytes.

## Results

### Gene expression of EGF receptor family members in PCMOs

**Figure 1:**
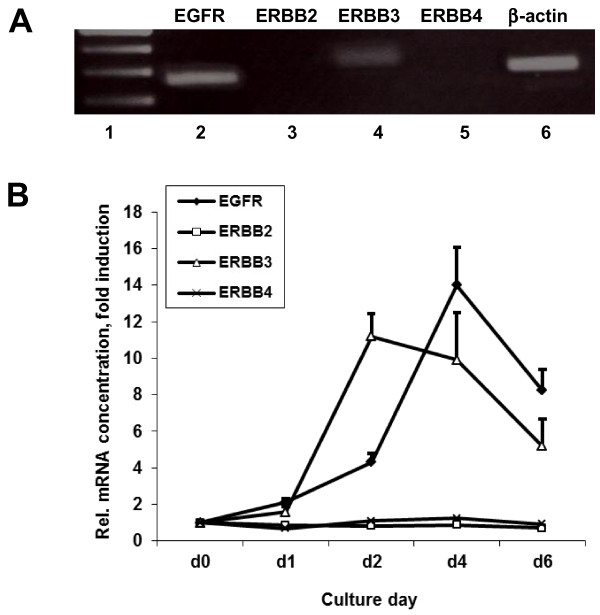
**Detection of different EGF receptor family members by RT-PCR in human blood monocytes.(A)** Standard endpoint RT-PCR of EGFR (lane 2), ERBB2 (lane 3), ERBB3 (lane 4), ERBB4 (lane 5), and β-actin as control (lane 6) in PCMOs. RNA was isolated from day-4 PCMOs and reverse-transcribed. Amplification products of the resulting cDNA (using the primers listed in Table 1) were run on an agarose gel and stained with ethidiumbromide. A molecular weight marker (lane 1) was run in parallel to confirm the predicted band sizes. **(B)** QPCR analysis of the same EGF receptors in monocytes during the generation of PCMOs. Data shown are the mean ± SD of 3 separate experiments performed in duplicates.

We first sought to determine which EGF receptors are expressed in monocytes. For this purpose, RNA was isolated from monocyte cultures and processed for qPCR using primers for EGFR (also termed ERBB1), ERBB2, ERBB3, and ERBB4 as listed in Table 1. RT-PCR analysis of the four EGF receptors yielded a strong signal for EGFR and a weaker one for ERBB3 (Figure 1A). Since monocytes may be “contaminated” with lymphocytes, a negative control sample of highly purified lymphocytes was analyzed in parallel and shown to lack expression of both EGFR and ERBB3 (data not shown). This indicated that the amplification products for EGFR and ERBB3 were specifically derived from monocytes. Since the expression levels of some genes may differ during the development of PCMOs in culture, we isolated RNA from the developing PCMOs at different days of culture. The qPCR of these samples indicated that expression of both EGFR and ERBB3 initially increased during PCMO generation reaching a peak on the second day (ERBB3) and on the fourth day (EGFR) of culture and decreased thereafter (Figure 1B).

**Table 1:** Sequences of PCR primers

**Gene**	**Accession No.**	**Sense primer**	**Antisense primer**	**Product size (bp)**
**EGFR**	**NM_005228.3**	**ATGCTCTACAACCCCACCAC**	**GCCCTTCGCACTTCTTACAC**	**193**
**c-erbB2**	**NM_004448.2**	**CCCTCATCCACCATAACACC**	**GCCTGGCATTCACATACTCC**	**279**
**c-erbB3**	**NM_001982.3**	**TACTTGGAACGGGGTGAGAG**	**ACTCTGCCGTCCACTCTTGT**	**219**
**c-erbB4**	**NM_005235.2**	**AGTCAGTGTGTGCAGGAACG**	**CTCCAGAGGCAGGTAACGAA**	**224**
**Nanog**	**NM_024865**	**GATTTGTGGGCCTGAAGAAAACT**	**AGGAGAGACAGTCTCCGTGTGAG**	**79**
**GAPDH**	**NM_002046**	**TTGCCATCAATGACCCCTTCA**	**CGCCCCACTTGATTTTGGA**	**174**
**β-actin**	**NM_031144**	**GATATCGCTGCGCTCGTC**	**TCCATATCGTCCCAGTTGG**	**239**
**ANAPC2**	**NM_013366.3**	**CCAGTACAGGCGGTGATCTT**	**GCTCTCGTCGTCACTGTCAA**	**228**
**ABL-1**	**NM_005157.4**	**AACACCCTAACCTGGTGCAG**	**CAAGTGGTTCTCCCCTACCA**	**248**
**CDC2 (CDK1)**	**NM_001786.4**	**GGGGTCAGCTCGTTACTCAA**	**GATGCTAGGCTTCCTGGTTTC**	**225**
**CDK4**	**NM_000075.2**	**CTGACCGGGAGATCAAGGTA**	**AGCCAGCTTGACTGTTCCAC**	**224**
**CDK6**	**NM_001145306.1**	**TCCCAGGAGAAGAAGACTGG**	**GGTCCTGGAAGTATGGGTGA**	**198**

### EGF promotes proliferation during PCMO production

**Figure 2:**
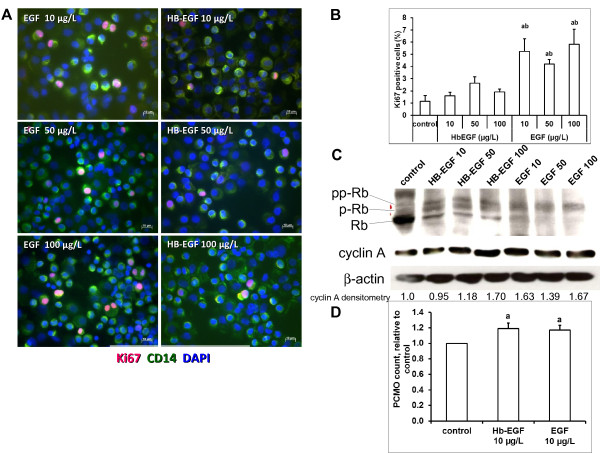
**EGF and HB-EGF increase the number of mitotically active cells in PCMO cultures.(A)** immunofluorescence of Ki67 expression (red) in PCMO cultures treated for 4 days with the indicated concentrations of either EGF or HB-EGF. PCMOs were immunostained with CD14 (green) as a monocyte-specific marker. Nuclei were stained with DAPI (blue). **(B)** Quantification of Ki67 expressing PCMOs after treatment with EGF or HB-EGF. Data (N = 3) are expressed as mean ± SEM. Statistical analysis: “a” denotes a significant difference from the control; “b” denotes a significant difference from the corresponding values of HB-EGF. **(C)** Immunoblotting of the retinoblastoma protein and cyclin A in PCMO cultures treated for 4 days with the indicated concentrations (in μg/L) of either HB-EGF or EGF. The housekeeping protein β-actin was detected as a loading control. Densitometric analysis of cyclin A and β-actin bands was performed using NIH ImageJ software (version 1.45). The indicated values below the blots represent the normalized signal intensities for cyclin A relative to untreated control cells set at 1. **(D)** PCMO cell counts after 4 days of culture with 10 μg/L of either EGF or HB-EGF (N = 3). Statistical analysis: “a” denotes a significant difference from the control.

Next, we examined the effect of EGF and HB-EGF on the proliferation of PCMOs (Figure 2). For this purpose, cells were cultured for 4 days in PCMO medium containing EGF or HB-EGF at different concentrations. Cells were prepared for immunofluorescence using Ki67 antibody as a proliferation marker and CD14 as a monocyte marker. The results showed a higher number of Ki67/CD14 double-positive cells in both EGF and HB-EGF-treated cultures (Figure 2a). However, quantification of these cells showed that the HB-EGF but not the EGF effect closely missed statistical significance (Figure 2B). No statistically significant differences of Ki67/CD14-positive cell counts were observed among different concentrations of the same treatment. These data clearly show that the addition of EGF enhanced the proliferative activity of monocytes in PCMO generation medium. EGF-induced proliferation temporally correlated with cell cycle activation. In order to investigate whether EGF-induced proliferation was associated with the expression of specific cell-cycle regulatory genes, we treated monocytes with different concentrations of EGF or HB-EGF and performed qPCR analysis as described in the Methods section. As seen in Table 2, both EGF and HB-EGF up-regulated the expression of ABL, ANAPC2, CDC2, CDK4, and CDK6, each of which is involved in different stages of the cell cycle.

**Table 2:** Expression of cell cycle genes during the development of PCMOs cultured in the presence of HB-EGF or EGF

	**ABL**	**ANAPC2**	**CDC2**	**CDK4**	**CDK6**
**Hb-EGF 10**	1.40 ± 0.19^**a**^	1.74 ± 0.62	1.88 ± 0.74	1.53 ± 0.33	2.42 ± 1.10
**Hb-EGF 50**	1.26 ± 0.15^**a**^	1.40 ± 0.45	1.81 ± 0.77	1.56 ± 0.33	2.09 ± 0.80
**Hb-EGF 100**	1.48 ± 0.43	1.63 ± 0.66	2.23 ± 0.95	1.47 ± 0.27	2.35 ± 1.06
**EGF 10**	2.24 ± 0.18^**ab**^	1.78 ± 0.43	3.01 ± 1.05^**ab**^	1.57 ± 0.17	2.47 ± 0.96
**EGF 50**	1.69 ± 0.20^**ab**^	1.70 ± 0.42	1.99 ± 0.42^**a**^	1.39 ± 0.10	2.15 ± 0.87
**EGF 100**	1.74 ± 0.30^**ab**^	1.60 ± 0.38	2.02 ± 0.50^**a**^	1.53 ± 0.14	2.38 ± 0.93
***ANOVA =***	***0.0033***	***0.0748***	***0.036***	***0.261***	***0.0756***

RNA was isolated from PCMOs after 4-day culture with or without EGF or HB-EGF (10, 50, 100 μg/L) and transcribed to cDNA. QPCR was applied using primer pairs listed in Table 1. Data are presented as mean ± SEM of N = 4 and represent the fold-change in comparison with control PCMOs (cultured without HB-EGF or EGF), the values of which were considered as 1. Statistical analysis: a = significantly different from the control, b: significantly different from the corresponding HB-EGF value.

The retinoblastoma protein (pRb) plays a pivotal role in the negative control of the cell cycle and prevents the cell from replicating damaged DNA by blocking progression through G1 into S phase. Its inhibitory role on cell cycle progression is carried out in the hypophosphorylated state, while phosphorylation inactivates pRb [19]. We have analysed the phosphorylation state of pRb in PCMOs generated in the presence of either EGF or HB-EGF (Figure 2C). The results show that treatment with HB-EGF increased the phosphorylation of pRb, while EGF caused its hyperphosphorylation. In control cells, however, only the active non-phosphorylated form was present (Figure 2C).

We have also investigated cyclin A protein in the same samples. Cyclin A defines control points of the cell cycle. It binds both CDK2 and CDC2 giving rise to two distinct cyclin A kinase activities, one appearing in S phase and the other one in G2 phase [20]. Immunoblotting indicated an increase in cyclin A expression upon treatment of PCMOs with 50 and 100 μg/L HB-EGF and with all three concentrations of EGF (Figure 2C).

Finally, we performed cell counting of PCMOs cultured for 4 days with either 10 μg/L EGF or HB-EGF (Figure 2D). The results demonstrated a moderate but significant increase over the control in total cell counts after both treatments. No difference was observed between the two treatments. Together, the data show that EGF and HB-EGF are suitable tools to expand the total cell number of PCMOs and that this largely occurs through an increase in the mitotic/cell cycle activity of monocytes.

### EGF treatment attenuates expression of p47^phox^ and enhances expression of Nanog in PCMOs

**Figure 3:**
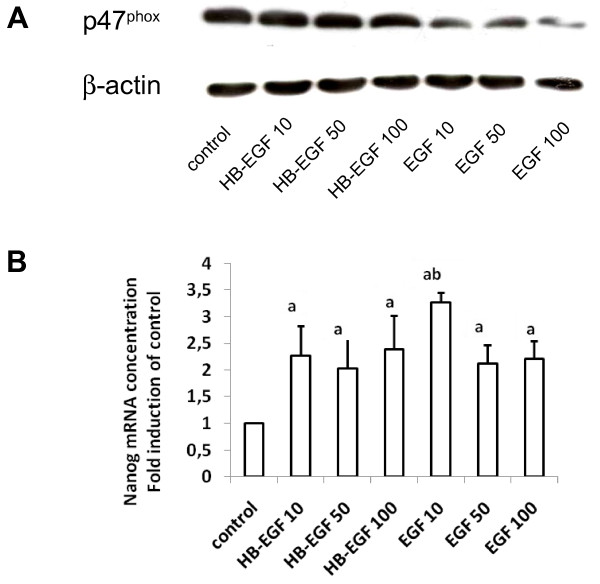
**Regulation of Nanog and p47**^**phox**^**expression by EGF or HB-EGF in PCMOs.(A)** Measurement of Nanog expression by qPCR in PCMOs treated for 4 days with the indicated concentrations (in μg/L) of HB-EGF or EGF. Data (mean ± SEM, N = 4) were normalized to the expression level of GAPDH. ANOVA: p < 0.01; a = all values of EGF and HB-EGF are significantly higher than that of the control; b = significant difference between EGF and the corresponding HB-EGF value. **(B)** Immunoblot analysis of p47^phox^ in day-4 PCMOs treated with either HB-EGF or EGF at the indicated concentrations. The β-actin protein was detected as a loading control.

During the generation of PCMOs, monocytes downregulate markers of differentiation, e.g. p47^phox^ an essential subunit of the reactive oxygen producing enzyme NAD(P)H oxidase and upregulate markers of pluripotency, e.g. Nanog [6]. We have examined the effect of EGF and HB-EGF on the expression of p47^phox^ by immunoblotting (Figure 3A) and on the expression of Nanog by qPCR (Figure 3B). The p47^phox^ protein levels were clearly lower on day 4 of culture which was particularly prominent in EGF-treated cultures (Figure 3A). No differences were observed between treatments with different concentrations of EGF. Both EGF and HB-EGF caused a more than 2-fold increase in the mRNA levels of *Nanog* (Figure 3B). Statistically significant differences were observed neither among EGF and HB-EGF treatments nor among different concentrations of each growth factor. The data suggest that EGF can enhance both the extent of dedifferentiation and pluripotency.

### MEK/ERK signaling drives proliferation in PCMOs and is superactivated by EGF and HB-EGF

**Figure 4:**
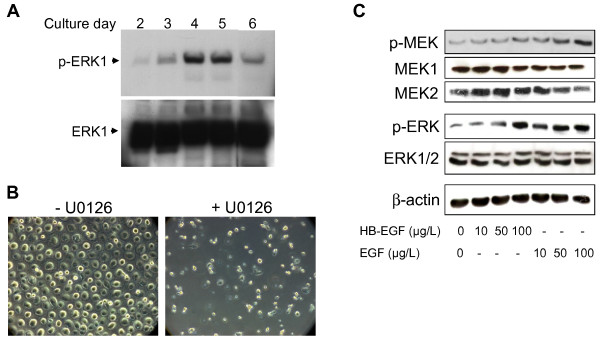
**MEK/ERK signaling drives proliferation in PCMOs and is superactivated by EGF and HB-EGF.(A)** M-CSF and Il-3 induce phosphorylation of ERK (pERK). Protein samples were obtained from PCMOs cultured for various days (as indicated) in standard PCMO differentiation medium. **(B)** Inhibition of ERK was sufficient to inhibit PCMO proliferation. Cells were cultured with either solvent (left) or the ERK inhibitor U0126 (right) for 6 days and images were taken. Original magnification: 50x. **(C)** HB-EGF and EGF induce phosphorylation of MEK and ERK. Phospho-MEK (p-MEK), MEK1/2, phospho-ERK (p-ERK), and ERK1/2 expression was examined by immunoblotting in lysates of day-4 PCMOs that have been treated with the indicated concentrations of either HB-EGF or EGF.

ERK and MEK activation is involved in M-CSF and EGF-induced proliferation of PCMOs. We have previously shown that during PCMO culture, a subset of monocytes resumes proliferation. To test whether this is associated with activation of MEK/ERK signaling, we performed immunoblot analysis of ERK activation (Figure 4A). ERK phosphorylation during PCMO generation peaked on day 3–4 of culture and this increase coincided with peak mitotic activity [2]. This suggested that ERK activation is causally involved in driving proliferation of monocytes/PCMOs. To test this more directly, we inhibited MEK1 with U0126 during PCMO culture and assessed the number of cells on day 6. The total number of cells was low, indicating that MEK/ERK signaling is crucial for PCMO proliferation (Figure 4B).

Since both EGF and HB-EGF are known to stimulate ERK activation, we reasoned that these agents may enhance proliferation by superactivating the MEK/ERK pathway. To test this prediction, PCMOs were generated in standard PCMO differentiation medium in the absence or presence of either EGF or HB-EGF and subjected to immunoblot analysis of phospho-MEK and phospho-ERK. The results indicated that both EGF and HB-EGF activated MEK and ERK and that the effect was concentration-dependent and more prominent in EGF-treated than in HB-EGF-treated PCMOs (Figure 4C).

### Effect of EGF and HB-EGF on NeoHepatocyte function

**Figure 5:**
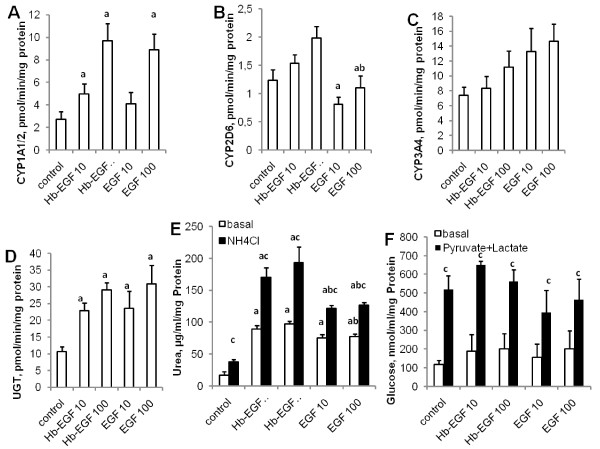
**Effect of EGF and HB-EGF treatment during PCMO culture on NeoHepatocyte function.** PCMOs treated with the indicated concentrations (in μg/L) of EGF or HB-EGF were cultured in hepatocyte conditioning medium for 2 weeks before subjecting them to analysis of cytochrome P450 (CYP) isoforms 1A1/2 **(A)**, 2D6 **(B)**, and 3A4 **(C)**, the phase II enzyme UDP-glucuronosyl transferase **(D)**, urea metabolism **(E)**, and glucose metabolism (F). Methodologic details are given in the Methods section. Data are presented as mean ± SEM of N = 4. Statistical analysis: Single-Factor ANOVA: p < 0.01 for A, B and D; ANOVA (2-Factor with replication) for E: p < 0.001 between different EGF/HB-EGF treatments; p < 0.001 between basal and NH_4_Cl-treatments; for F: p < 0.001 between basal and pyruvate + lactate treatments; a = significantly different vs. control; b = vs. corresponding HB-EGF value; c = vs. basal value.

Ideally, a modification of the PCMO generation procedure should not only enhance proliferation but also the stem cell features of PCMOs in a way that the resulting NeoHepatocytes become more hepatocyte-like. We therefore tested whether adding EGF and HB-EGF to the PCMO generation medium would alter functional parameters of the Neoepatocytes. Control PCMOs and PCMOs generated in the presence of either EGF or HB-EGF were allowed to differentiate into NeoHepatocytes for 2 weeks and at the end of this period were analysed for hepatocyte-specific functions (Figure 5).

NeoHepatocytes, regardless of treatment, including the control, formed and secreted urea in similar amounts as under basic conditions. Addition of NH_4_Cl increased urea formation in all settings. However, it was higher in NeoHepatocytes obtained from PCMOs generated in the presence of HB-EGF. NeoHepatocytes regardless of treatment, including the control, all secreted glucose at similar rates. To measure the ability of the cells to perform gluconeogenesis, the Na-pyruvate-containing incubation buffer was supplemented with Na-L-lactate. Stimulation with pyruvate/lactate induced higher glucose secretion compared to non-stimulated cultures. As for urea, the effect was higher in NeoHepatocytes obtained from PCMOs generated in the presence of HB-EGF.

NeoHepatocytes exhibit phase I and II enzyme activities. However, levels were significantly lower compared to primary human hepatocytes [21] and could be enhanced by replacing the FCS with autologous serum [21]. We investigated the effect of EGF and HB-EGF on the activity of three different cytochrome P_450_ isoforms (1A1/2, 2D6, and 3A4) and a phase II enzyme (UDP-glucuronosyl-transferase). The activities measured in cells varied between the different treatments. CYP1A1/2 activity was similar in, NeoHepatocytes obtained from PCMOs treated with either EGF or HB-EGF, and the effect of both was concentration-dependent. CYP2D6 activity was higher in NeoHepatocytes obtained from PCMOs treated with HB-EGF than those treated with EGF. This situation was reversed for the activity of CYP3A4. The activity of the phase II enzyme UDP-glucuronosyl-transferase was similar for both treatments, but higher than that of the control.

## Discussion

Peripheral blood monocytes can be reprogrammed to generate a kind of stem cell-like cell (PCMO), which is sensitive to differentiation into hepatocyte-like cells [2,3]. In view of a potential clinical use of these cells in regenerative cell therapies such as treatment of end-stage liver diseases, the identification of factors capable of increasing the expansion of PCMOs/NeoHepatocytes is of great importance.

M-CSF and IL-3 present in the PCMO generation medium induce a proliferative response in a subset of monocytes through activation of MEK/ERK1/2 signaling (see Figure 4). Since this signaling pathway is also activated by EGF and HB-EGF and their receptors and is involved in the proliferation of many cell types [14-16], we reasoned that EGF should be able to further stimulate PCMO proliferation. In agreement with this assumption, we detected the expression of EGFR and ERBB3 in monocytes. The expression of both receptors gradually increased during monocyte/PCMO culture, suggesting a role for them in the process of PCMO generation. Activation of EGFR on monocytes has been reported to be required for monocyte activation and cellular motility [17]. EGF was found also to mediate monocyte chemotaxis and macrophage proliferation [18]. Taking advantage of the relative ability of monocyte subpopulations to undergo proliferation and generate PCMOs, we showed here that EGF and HB-EGF were able to increase total cell counts and the cells’ proliferative activity as assessed by Ki67 staining. With respect to Ki67 staining the HB-EGF effect did not reach statistical significance, which may be explained by donor-specific variations in the monocyte’s ability to respond to various treatments in culture (H.U., unpublished observation). The enhanced proliferation was accompanied by activation of cell cycle regulatory genes ANAPC2, ABL1, CDK4, CDK6, and CDC2. ANAPC2 plays an important role in the regulation of the G1/S and G2/M transitions while ABL1 regulates the S-phase and DNA replication. CDK4 and 6 participate in the G1/S transition and CDC2 in M-phase regulation. EGF was also previously reported to induce increased cyclin D1 expression in other systems [22]. Inhibition of some of the functional proteins such as ANAPC2 and CDC2 that form the anaphase promoting complex/cyclosome (APC/C) has been reported to induce cell cycle arrest at G2/M [23]. Thus, the induction of cell cycle arrest is associated with the down-regulation of genes involved in G1/S and G2/M transitions and an increase in the expression of these genes can lead to activation of the cell cycle. We confirmed these results by immunoblotting of pRb, which negatively regulates progression from G0 through to G1 and into S phase [24]. The results showed that treatment with EGF increased the pRb hyperphosphorylated form to a greater extent than HB-EGF which also showed a higher degree of phosphorylation than the control. pRb is normally hypophosphorylated in resting cells at G0 when proliferation is repressed. Upon activation of the cell cycle, appropriate signals lead to the subsequent activation of the cyclin D/CDK4 and 6, cyclin E/CDK2 and cyclin A/CDK2 complexes, which increasingly phosphorylate pRb during progression through G1. The pRb will be kept in a hyperphosphorylated (inactive) form until late in mitosis [25].

In contrast to GM-CSF [26], M-CSF and IL-3 induced tyrosine phosphorylation and activation of ERK in monocytes. Moreover, addition of the MEK inhibitor U0126 prevented M-CSF + IL-3-induced proliferation, strongly suggesting that MEK/ERK signaling drives the proliferative response of monocytes under standard culture conditions. In the present work, we demonstrated that addition of EGF or HB-EGF superactivated the MEK/ERK pathway and further increased proliferation. In other systems, the EGFR tyrosine kinase inhibitor Erlotinib, and U0126 completely inhibited EGF-induced proliferation [22]. Also, HB-EGF enhanced phosphorylation of Akt and ERK, implying a role for phosphatidylinositol 3-kinase (PI3K)/Akt and MEK/ERK signaling in HB-EGF-stimulated cell proliferation [16]. The PI3K inhibitors LY294002 and wortmannin, and the MEK/ERK inhibitors U0126 and PD98059, reduced HB-EGF-induced BrdU incorporation into cultures [16]. Taken together, it can be concluded that exposure of PCMOs to EGF or HB-EGF leads to activation of their receptors (ERBB1 and 3), the expression of which increases during PCMO culture, and subsequent activation of MEK/ERK. This extra input of ERK signaling is sufficient to further enhance PCMO proliferation beyond the level achieved with M-CSF + IL-3-induced ERK activation.

Our results showed that both EGF and HB-EGF activated cell proliferation-associated changes in PCMOs during their generation but that these effects were generally stronger for EGF. Nevertheless, treatment with both agents resulted in the same increase in total PCMO cell numbers. This suggests the possibility that HB-EGF, in addition to its growth-promoting function, exerts anti-apoptotic effects on PCMOs that contribute to cell expansion.

Interestingly, EGF and HB-EGF appear to enhance *de*differentiation of PCMOs (as assessed by a decrease in the expression of p47^phox^) and to increase pluripotency (as assessed by an induction of *Nanog*) [6]. We have previously characterized stem cell marker expression in PCMOs and have demonstrated similar expression profiles of *Nanog* and *Oct3/4* during PCMO generation [6]. Moreover, the expression of Nanog and Oct3/4 was paralleled by a global rise in histone H3 methylation on Lys-4, a marker of active chromatin, and coincided with peak sensitivity to hepatocyte-specific differentiation [6].

Functionally, both EGF and HB-EGF applied during PCMO generation improved the function of the resulting NeoHepatocytes when compared with those derived from control (standard) PCMOs. However, the present results demonstrated functional similarities of NeoHepatocytes obtained after PCMO treatment with either EGF or HB-EGF. When EGF and HB-EGF where compared for their potency to enhance the hepatocyte-specific functions of NeoHepatocytes no major differences were observed, although HB-EGF appears to be superior with respect to urea production, glucose metabolism and CYP2D6 activity, while EGF was superior in inducing CYP3A4 activity.

## Conclusions

The present data reveal that EGF and HB-EGF improve the proliferation of PCMOs by superactivating MEK/ERK signaling. Notably, however, both factors improve hepatocyte-specific functions of the resulting NeoHepatocytes which is an important issue when considering these cells for transplantation purposes. Based on these data, we suggest modifying the current protocol of PCMO generation by adding EGF or HB-EGF to the culture medium.

## Methods

### Generation of PCMOs

Human peripheral blood monocytes isolated from LRS chambers or buffy coats from healthy donors were isolated by density gradient centrifugation and further purified by adherence separation. Cells (1 x 10^5^/cm^2^) were allowed to adhere to tissue culture plastics for 1–2 h in RPMI 1640 medium containing 10% human AB serum (Lonza, Cologne, Germany), 2 mmol/L glutamine, 100 U/mL penicillin, and 100 μg/mL streptomycin (all from Invitrogen, Karlsruhe, Germany). Nonadherent cells were removed by aspiration, and the adherent monocytes were cultured for 4 days in *de*differentiation medium consisting of RPMI supplemented with 140 μmol/L 2-mercaptoethanol, 5 μg/L M-CSF, and 0.4 μg/L human IL-3 (both from R&D Systems, Wiesbaden, Germany)). In previous experiments these cells have been tested for purity by flow cytometry analysis of CD45 and CD14, typically yielding a purity of 70-80% [2]. Either EGF or HB-EGF (both from R&D Systems) was added to the *de*differentiation medium at various concentrations (10, 50, or 100 μg/L). The MEK inhibitor U0126 was purchased from Calbiochem/Merck (Darmstadt, Germany) and dissolved in dimethyl sulfoxide.

### Differentiation of PCMOs into NeoHepatocytes

After 4 days of culture in *de*differentiation medium PCMOs were cultured for 2 weeks with hepatocyte conditioning medium (RPMI 1640 medium containing 3 μg/L fibroblast growth factor-4 (FGF-4, R&D Systems) and 10% FBS) for differentiation into NeoHepatocytes [2]. The medium was changed every 3 days. Cells were then subjected to analysis of hepatocyte function.

### Immunofluorescence

PCMOs were washed with PBS, centrifuged and diluted with PBS containing 1% BSA, centrifuged at maximal speed for 3 min using the Cytospin 4 centrifuge (Thermo Scientific) and kept in −20°C until needed. For proliferative cell staining, slides were fixed in 1% paraformaldehyde, blocked for 1 h and then incubated with anti-human CD14 antibody (BD Biosciences, Heidelberg, Germany) at room temperature for 2 h and Alexafluor 488–labeled secondary antibody (Invitrogen) for 1 h. After washing, cells were permeabilized using 0.5% triton X-100 and incubated overnight with the anti-human Ki67 (BD Pharmingen) at 4°C followed by Alexafluor 555-labeled secondary antibody (Invitrogen). Ki67-positive cells were counted double-blind by two investigators in at least 4 visual fields per slide, repeated for all experiments (N = 4) and related to the total cell count of CD14-positive monocytes in the same field.

### RNA isolation and quantitative RT-PCR

Total RNA isolation from PCMOs, human peripheral blood monocytes and autologous lymphocytes (purified by elutriation as described earlier [6]) was performed using the GeneJet purification kit (Fermentas, St. Leon-Rot, Germany). To assure absence of genomic DNA, all RNA samples were treated with DNase I, and primers spanning multiple exon-intron boundaries were used. For reverse transcription, 1 μg of the total RNA was reverse transcribed to first strand complementary DNA using the High-Capacity reverse transcription kit (Applied Biosystems, Darmstadt, Germany). Gene expression was quantified by standard endpoint RT-PCR and standard real-time RT-PCR (qPCR) on an iCycler (Bio-Rad, Munich, Germany) and analyzed by agarose gel electrophoresis and iCycler iQ Real-Time Detection System software (Bio-Rad), respectively. The thermal cycling program was 10 min at 95°C for enzyme activation, denaturation for 15 s at 95°C, 60 s annealing at 60°C, and 60 s extension at 72°C. A dissociation curve was performed for each product to assure the absence of primer dimers or unspecific products. Primers used in the present study are listed in Table 1. Relative quantification was performed by ΔΔCt method. To normalize expression data, amplification of the housekeeping gene GAPDH was used as an internal control.

### Western blotting

Following 4 days of PCMO generation, cells were thoroughly washed with PBS to remove non-adherent cells (mainly T lymphocytes) and lysed using PhosphoSafe lysis buffer (Novagen/Merck). Cell lysates were separated by electrophoresis prior to transfer to PVDF membranes (Immobilon P). Membranes were then probed with primary antibodies and immunoreactive bands were detected by chemiluminescence. Primary antibodies used were MEK1 (C-18), MEK2 (C-16), p-MEK1/2 (Ser218/Ser222), ERK1 (C-16), p-ERK (Tyr 204) (all from Santa Cruz Biotechnology, Heidelberg, Germany), anti-human pRb (BD Pharmingen), and β-actin (Sigma, Deisenhofen, Germany). Secondary antibodies were obtained from GE Healthcare (Buckinghamshire, UK).

### Analysis of NeoHepatocyte function

Urea measurement: To remove residual urea from the culture medium, cells were washed twice with DPBS. To determine basal levels of urea formed, cells were incubated with DPBS (1 mM MgCl_2_, 1 mM Na-pyruvate) for 24 h. To measure the ability of the cells to metabolize ammonium, the buffer was supplemented with 5 mM NH_4_Cl ± 1 mM ornithine. Supernatant (80 μl) was incubated with 60 μl 0.0002% O-phthaldehyde solution (0.03% Brij-35, 7.4 % H_2_SO_4_) and 60 μl NED reagent [0.0006% N-(1-naphthyl)ethylenediamine dihydrochloride, 0.5% boric acid, 0.03% Brij-35, 22.2% H_2_SO_4_ for 2 h at 37°C. Absorbance was measured at 505 nm and compared to standard samples [21].

Glucose measurement: Cells were washed three times with DPBS before incubation for 24 h with DPBS (30 mM KCl, 1 mM MgCl_2_, 1 mM Na-pyruvate ± 10 mM Na-L-lactate). Supernatant (100 μl) was incubated with 150 μl GLOX solution (250 mM Tris, 0.2 mM EDTA, 0.04% glucose-oxidase, 0.007% peroxidase, 0.01% O-dianisidine, pH 8.0) for 2 h at 37°C. Absorbance was measured at 420 nm and compared to standard samples.

Phase I and II Enzyme activity assays: Fluorescence-based cytochrome P450 assays were performed by incubation of intact cells with selected substrates as reported [21]. Briefly, cells cultured on a 96-well plate were serum starved (RPMI 1640 medium without supplements) overnight prior to measurement. For measurement the medium was replaced with 100 μl reaction buffer (plain RPMI 1640 medium containing the fluorogenic substrates: 25 μmol/L 7-ethoxy coumarin for CYP1A1/2, 10 μmol/L AMMC (3-[2-(N,N-diethyl-N-methylamino)ethyl]-7-methoxy-4-methylcoumarin) for CYP2D6, 10 μmol/L BFC (7-benzyloxy-4-trifluoromethylcoumarin) for CYP3A4 and 100 μmol/L 4-methylumbelliferon as a substrate for UDP-Glucuronosyl-transferase). Fluorescence was measured every 10 min over a period of 2 h with a microplate reader (BMG Labtech, Offenburg, Germany). Afterwards cells were fixed for protein quantification by sulforhodamine B (SRB) staining as previously described [27]. Results are given as pmol of fluorescent product formed (phase I enzyme activities) or fluorescent substrate reduced (for phase II enzyme activities) per minute normalized to total protein content in mg.

### Statistical analysis

All samples were measured in duplicates. Values were expressed as mean ± SEM. with N = 4 in all experiments. Group statistical comparisons were performed by one-way or two-way analysis of variances (ANOVA) followed by Mann–Whitney multi-range analysis as a post-hoc test. The *p* values were shown in the Results section A statistical difference was considered significant if *p* < 0.05.

## Abbreviations

CDK, Cyclin-dependent kinase; EGF, Epidermal growth factor; ERK, Extracellular signal-regulated kinase; FBS, Fetal bovine serum; GM-CSF, Granulocyte macrophage colony-stimulating factor; h, Hour; HB-EGF, Heparin-binding epidermal growth factor; IL-3, Interleukin-3; M-CSF, Macrophage colony stimulating factor; PBS, Phosphate-buffered saline; PCMOs, Programmable cell(s) of monocytic origin; pRb, Retinoblastoma protein; s, Second; SDS-PAGE, Sodium dodecyl sulfate polyacrylamide gel electrophoresis.

## Competing interests

The authors have no competing interests to declare.

## Authors’ contributions

AH, FF, and HU designed the study and drafted the manuscript; AH, SE, and AN coordinated and conducted the experiments. All authors read and approved the final manuscript.
